# A Bioelectronic Platform Modulates pH in Biologically Relevant Conditions

**DOI:** 10.1002/advs.201800935

**Published:** 2019-01-28

**Authors:** Xenofon Strakosas, John Selberg, Xiaolin Zhang, Noah Christie, Peng‐Hao Hsu, Adah Almutairi, Marco Rolandi

**Affiliations:** ^1^ Department of Electrical Engineering University of California Santa Cruz Santa Cruz CA 95064 USA; ^2^ UCSD Center of Excellence Department of NanoEngineering Jacobs School of Engineering University of California San Diego 9500 Gilman Dr. La Jolla CA 92093 USA

**Keywords:** bioelectronics, pH, protons

## Abstract

Bioelectronic devices that modulate pH can affect critical biological processes including enzymatic activity, oxidative phosphorylation, and neuronal excitability. A major challenge in controlling pH is the high buffering capacity of many biological media. To overcome this challenge, devices need to be able to store and deliver a large number of protons on demand. Here, a bioelectronic modulator that controls pH using palladium nanoparticles contacts with high surface area as a proton storage medium is developed. Reversible electronically triggered acidosis (low pH) and alkalosis (high pH) in physiologically relevant buffer conditions are achieved. As a proof of principle, this new platform is used to control the degradation and fluorescence of acid sensitive polymeric microparticles loaded with a pH sensitive fluorescent dye.

Bioelectronics couples electronics and biology with devices that transduce electronic to biological signals and vise‐versa, for the purpose of diagnosis and treatment. Numerous diagnostic sensors exist including for glucose,^1^, ^2^ lactate,^3^ neurotransmitters,^4^ electrophysiological measurements,^5^, ^6^ and barrier tissue integrity.^7^, ^8^ Electroceutical devices for treatment of disease include metal electrodes implanted in the brain to stimulate damaged neurons (deep brain stimulation),^9^, ^10^, ^11^ organic electronic devices that electrophoretically deliver small ions (K^+^, Ca^2+^) and neurotransmitters (GABA^+^) to the extracellular space (iontronics).^12^, ^13^, ^14^, ^15^

Among potential ions, protons (H^+^) play a vital role in biological processes. H^+^ currents can be found in proton‐gated ion channels^16^, ^17^ and proton pumps^18^; often these currents are coupled to the movement or generation of other chemical species such as in oxidative phosphorylation.^19^ The H^+^ concentration (pH) within a cell affects enzymatic activity,^20^, ^21^ gene expression,^22^ and is indicative of healthy cell function.^23^ pH regulation in the central nervous system maintains healthy neuronal function,^24^ and pH‐affects neuronal excitability. Acidosis plays a role in self‐termination of epileptic seizures.^25^ A common trait of cancer cells is a decrease of the extracellular pH due to overproduction of lactic acid; acid sensitive drug delivery vesicles use this mechanism to locally visualize and treat cancer cells and tissue.^26^, ^27^ Along with iontronics, bioprotonics use specifically H^+^ as their main charge carriers. To date, devices with conducting polymers and ion selective membranes, have demonstrated the transfer of H^+^ between different electrolytes and injection of H^+^ in common electrolytes and localized acidosis.^28^ To induce alkalosis and increase pH, a bioelectronic device needs to specifically absorb H^+^ ions without changing the concentration of other ions in solution. Palladium (Pd) contacts are able to specifically transfer H^+^ to and from solution and proton conducting polymers exploiting the specific and reversible Pd/palladium hydride (PdH*_x_*) reaction.^29^, ^30^ In this fashion, our group has demonstrated localized acidosis and alkalosis to control the rate of enzymatic reactions and bioluminescence.^29^, ^30^ However, these devices lack the dynamic range of the iontronic H^+^ pumps that allows to lower the pH in solutions with high buffering capacity such as brain fluid ($\beta \;=\;2.3\;x\;[{\mathrm{HCO}}_{3}^{-}]\;$, ≈ 60 × 10^−3^ m).^24^ Here, we combine the large dynamic range of iontronic type H^+^ pumps with the specificity of Pd/PdH*_x_* contacts^28^, ^31^ in a pH modulator that is able to induce both acidosis and alkalosis in solutions with high buffering capacity (**Figure** **1**).

**Figure 1:**
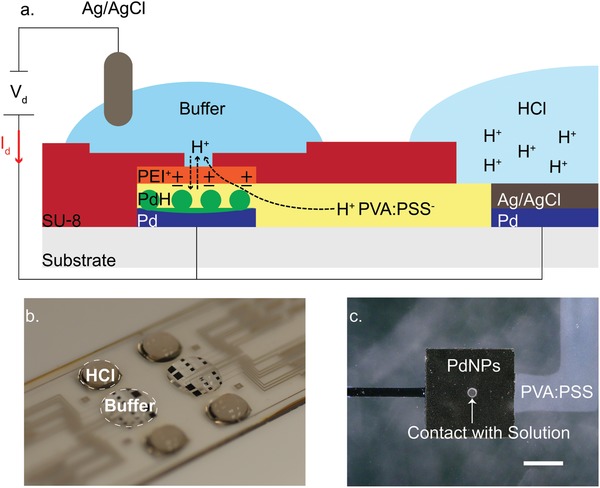
a) Schematic of pH modulator, and operating principle of bioelectronic pH modulator. Two electrolyte chambers are connected with an H^+^ conducting membrane. A high [H^+^] solution (right) is at the interface with a modified Pd/Ag/AgCl. A buffer solution (left) is at the interface with PdNPs and an Ag/AgCl pellet immersed in solution. Application of *V*_d_ = 1 V between PdNPs‐Pd/Ag/AgCl (working electrode) and Ag/AgCl pellet (reference electrode) decreases the buffer potential with respect to reservoir, and H^+^ are transferred from the reservoir (right) into the buffer solution and thus decrease its pH. Application of *V*_d_ = −1 V increases the potential of the buffer solution, and H^+^ are transferred and stored into the PdNPs, thus the pH of the buffer solution is increasing. b) Perspective photo of the device showing the reservoir loaded with acid (HCl) on top of the Pd/Ag/AgCl electrode and the active area with buffer on top of the PdNPs electrodes. c) Optical image showing a close up of a PdNPs contact, the proton bridge, and SU8 pore (40 µm) at the PdNPs/solution interface, scale bar 200 µm.

The pH modulator was fabricated in a four step photolithography (Figure S1, Supporting Information). The pH modulator involves independent electrolyte chambers connected by a proton conducting bridge. One electrolyte, consisting of 0.5 m HCl is used as a proton source (reservoir) and is in direct contact with a Pd contact modified with Ag/AgCl. The other electrolyte (target) is a buffer solution in which we induce pH modulation. The buffer electrolyte is in direct contact with 16 square Pd nanoparticle (PdNP) contacts (12 electrodes with an edge of 400 µm, and four electrodes with an edge of 1 mm) and an Ag/AgCl pellet (Figure 1a,b). The contacts are used as multiple inlets for H^+^ to enter the buffer solution in order to modulate the pH in a homogenous way. A positively charged membrane was patterned on top of the Pd contacts and between the cationic bridge and the buffer solution in order to reduce undesirable H^+^ diffusion from the reservoir to the target electrolyte. Finally, an epoxy resist (SU8) was used to separate both the target solution and proton reservoir, insulating the H^+^ bridge and the Pd interconnects from external stimuli. The SU8 allows for an inlet pore of a tunable size between buffer and PdNPs (Figure 1c).

The pH modulator uses one input voltage (*V*_d_) between the Ag/AgCl pellet, which is used as a reference electrode (RE), and the PdNPs contacts in order to increase or decrease the pH. The PdNPs contacts are held at the same potential with the modified Pd (electrodeposited Ag/AgCl on top of the Pd) contact in the reservoir (short circuit) (Figure 1a). When a negative *V*_d_ = −1 V is applied between the PdNPs contacts and the Ag/AgCl RE pellet, H^+^ are transferred from the buffer solution into the Pd contacts, forming PdH*_x_*, with *x* being the atomic ratio of H to Pd and its maximum value can reach to 0.6–0.7.^32^, ^33^ This results in a reduction of the proton concentration in the buffer electrolyte, thus, increase of its pH.^29^ When the *V*_d_ is reversed (*V*_d_ = 1 V), H^+^ are transferred from the reservoir solution, through the cationic selective bridge (Figures S3 and S4, Supporting Information), to the buffer solution and decrease its pH.

In the target electrolyte, Pd contacts were modified with PdNPs through electrodeposition, in order to increase the surface area, capacitance, and the ability to transfer H^+^ and modulate pH (**Figure** **2**a). A negative voltage (vs Ag/AgCl) applied to the Pd contact as working electrode and a Pd wire as counter electrode deposits PdNPs in the presence of a palladium nitrate (PdNO_3_) solution. We optimized the process by measuring the impedance of the contact at the end of the deposition. For a Pd contact of 400 µm, applying a voltage *V* = −0.6 V for 0.1 s deposits PdNPs and increases the surface area by ≈20‐fold, as calculated from an equivalent circuit R(R//C) by using electrochemical impedance spectroscopy (Figure 2b). Scanning electron microscopy (SEM) images show homogeneous PdNPs on top of Pd (Figure S2, Supporting Information). For extensive deposition times, PdNPs start aggregating thus reducing the surface area. We characterized the modified PdNP contacts with cyclic voltammetry (CV) in a (4‐(2‐hydroxyethyl)‐1‐piperazineethanesulfonic acid) HEPES buffer solution. We cycled the voltage of the PdNP contact between positive and negative values, versus an Ag/AgCl reference, for multiple cycles to monitor capacitive ionic currents and currents from interfacial reactions of the working electrode. In the case of Pd and PdNPs in HEPES, the main interfacial reaction between −1.1 and 0 V is the absorption and release of H^+^. In the PdNPs the number of H^+^ transferred at the PdNPs/electrolyte is 10 times higher than for the pristine Pd contact as measured by the current in the CV. (Figure 2c). Threshold voltages for H^+^ are also lower for PdNP. Absorption of H^+^ into PdNPs begins at *V* = −0.73 V compared to planar Pd at *V* = −0.8 V. The release of H^+^ starts at *V* = −0.4 V compared to *V* = −0.3 V for planar Pd. Additionally, the slope of the current d*I/*d*V = G* for the transfer of H^+^ into the electrolyte is ≈20 times larger than for planar Pd. We believe that the voltage shift to lower potentials originates from the increased surface area of the Pd contact when coated with PdNPs, the increased surface area exposes additional Pd sites for H^+^ transfer with a net effect of lowering the overpotential required for the hydrogen oxidation reaction. As a result of the more efficient H^+^ transfer characteristics, the PdNPs contacts modulate the pH in buffer conditions at least 10 times more than a planar Pd contact. A square PdNPs contact of 250 µm edge size induces a deltapH (ΔpH) ≈ 0.5 in 1 µL of a HEPES (β = 23 × 10^−3^ m), compared to a negligible change in pH induced by planar Pd (Figure 2d).

**Figure 2:**
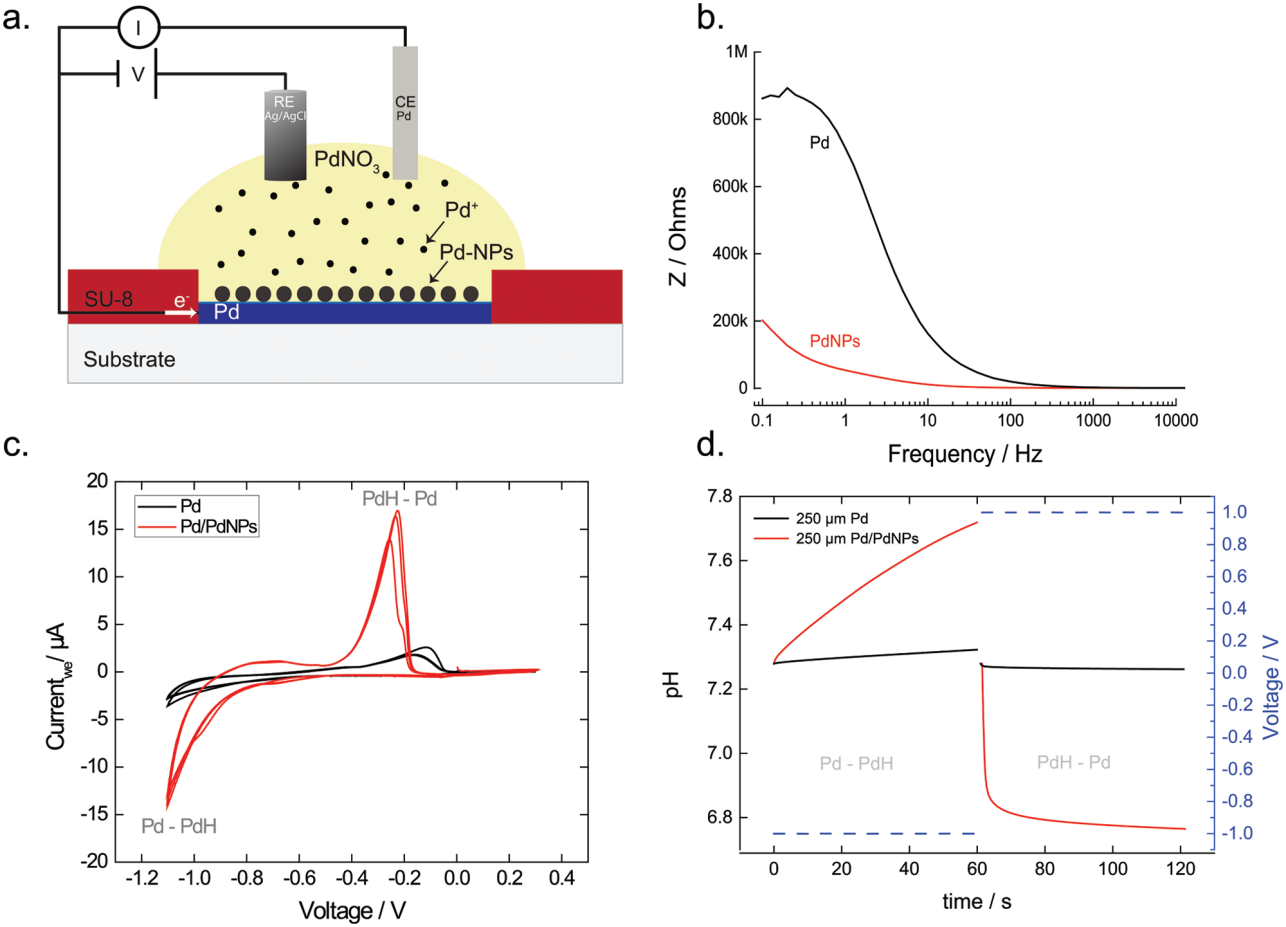
Electrodeposition of PdNPs on top of Pd. a) Schematic showing the electrodeposition. In the presence of PdNO_3_, a negative voltage of *V* = −0.6 versus Ag/AgCl reduces the Pd cations on top of Pd and creates PdNPs. b) Impedance measurements of PdNPs modified contact and a Pd contact. The PdNPs contact exhibits a lower impedance, which means higher capacitance/surface area. c) CV of a 400 µm Pd and PdNPs modified contacts in HEPES buffer. The CV shows the voltage regimes of H^+^ transfer into the (Pd‐PdH) and H^+^ transfer to the electrolyte (PdH‐Pd). Additionally, the current magnitude for PdNPs electrode is ≈10x higher. d) pH modulation of 1 µL HEPES from a 250 µm PdNPs and Pd contact.

**Figure** **3** shows how the device modulates the pH toward acidic and basic conditions. Prior to the measurements, extensive voltage cycling between target and reservoir in the presence of HCl 0.1 m in di‐water was performed in order to remove Na^+^ ions from the PVA:PSS H^+^ bridge. Additionally, the Pd/PdH contact is selective to H^+^ and if Na^+^ were transferred they would create charged layer at the contact resulting in a reduced current. During measurements, the PdNPs contacts are fixed (short circuit) at the same potential with the Pd/Ag/AgCl contact at the reservoir solution. To operate the device as a H^+^ sink (increasing pH), a negative voltage (*V*_d_ = −1 V) is applied between the PdNPs‐Pd/Ag/AgCl and the Ag/AgCl RE pellet. At the pH of 7.4 and potential difference, H^+^ are transferred from the buffer solution into the PdNPs contacts. The current has a transient capacitive behavior, which is typical for Pd contacts during H^+^ absorption and it is then stabilized to a constant negative value (Figure 3a).^29^ For every H^+^ that enters the PdNPs contact, a Cl^−^ is transferred from the buffer into the Ag/AgCl in order for the solution to maintain charge neutrality. In the H^+^ delivery mode (decreasing pH), a positive voltage (*V*_d_ = 1 V) is applied between the PdNPs‐Pd/Ag/AgCl and the Ag/AgCl pellet. During this step, H^+^ travel from the reservoir to the buffer solution decreasing its pH.^24^ The current is mainly resistive with a small initial capacitance, and a magnitude of 30–40 µA (Figure S5, Supporting Information). The capacitive portion of the current can be attributed to negatively charged ions, mainly OH^−^, that adsorb on the PdNPs a positive potential (Figure S6, Supporting Information).^34^, ^35^ A much higher initial capacitive current occurs when the PdNPs contacts are preloaded with H^+^ (Figure 3a and Figure S5, Supporting Information).^32^ Consecutive pulses between *V*_d_ = 1 V and *V*_d_ = −1 V for 60 s (Figure 3a) exhibit reproducible current behavior, and consequent pH oscillations (Figure S5, Supporting Information). Figure 3b shows the pH changes for a 50 µL of HEPES buffer upon an applied *V*_d_ = 1 V and *V*_d_ = −1 V for 120 s. The pH changes were calculated from the current by using the Henderson–Hasselbalch equation for the specific buffer.^18^ The calculated pH is in agreement with the experimental measurements of pH that were performed with a micro pH meter. The pH changes in Figure 3b are calculated for the overall volume of the HEPES buffer. Transiently, the pH changes are largest close to the Pd contacts, and subsequently propagate by following diffusion reaction phenomena.

**Figure 3:**
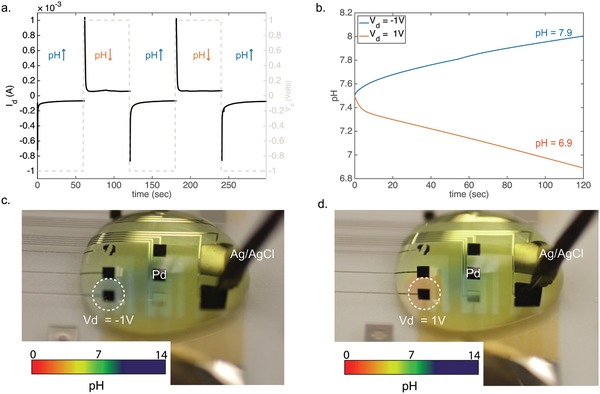
Protonic pH modulating circuit in action a) to increase the buffer pH, a negative voltage *V*_d_ = −1 V is applied between PdNPs‐Pd/Ag/AgCl contacts and the Ag/AgCl pellet in the buffer electrolyte transfers the H^+^ into the PdNPs. The resulting *I*_d_ has a typical Pd‐PdH behavior with steady state of −40 µA. To decrease the pH, a positive voltage *V*_d_ = 1 V between the PdNPs‐Pd/Ag/AgCl and the Ag/AgCl electrode in the buffer electrolyte transfers the protons from the reservoir into the buffer electrolyte. b) Calculated pH for *V*_d_ = −1 V (blue) for 120 s and *V*_d_ = 1 V (acidic) for 120 s on HEPES buffer. c) Optical image showing basic pH (blue color) in HEPES buffer with a pH indicator from an individual PdNPs contact. d) Optical image showing acidic pH (red color) in HEPES buffer with a pH indicator from an individual PdNPs contact. The pH color scale was provided by universal pH indicator documentation.

Figure 3c,d show localized pH changes that are induced by individual PdNPs contacts, in a buffer solution loaded with a universal pH indicator. A basic pH is induced on top of a PdNPs contact (blue), when *V*_d_ = −1 V (Figure 3c). The pH close to the PdNPs contact is higher compared to bulk of the solution, this pH gradient will slowly diffuse throughout the bulk solution until equilibrium is reached. When the voltage is reversed (*V*_d_ = +1 V), the pH locally changes from basic (blue) to acidic (red) on top of the Pd contact (Figure 3d). This illustrates the contribution of a PdNPs contact that is preloaded with H^+^ toward pH modulation which is caused by the release of stored H^+^ back into the electrolyte and the adsorption of OH^−^ on the PdNPs surface. By controlling parameters such as the duration of the input voltage and number of PdNPs contacts, the pH modulator can achieve pH changes in biological buffers. These changes are initially localized close to the contact and eventually affect the entire solution after diffusion of the H^+^ or OH^−^ species.

To demonstrate the ability of our device to control pH in physiologically relevant conditions, we used acid‐sensitive microparticles loaded with a pH sensitive dye fluorescein diacetate (FDA) in buffered conditions as a proof‐of‐concept target. By switching the environment between basic and acidic conditions we observed the degradation of the microparticles, release of FDA, followed by hydrolysis in basic conditions to fluorescein yielding an increase of the dye's fluorescence (**Figure** **4**a). The microparticles, which are based on dextran tagged with rhodamine, were synthesized as previously described.^27^ The microparticles, with average size of 2 µm and concentration of 1 mg mL^−1^, were dissolved in HEPES buffer of pH 7.4 and then were exposed to four conditions; control groups in solutions set at physiological pH 7.4, acidic pH 6, and basic pH 8, as well as a device modulated acidic then basic treatment switching the pH from neutral to acidic then to basic.

**Figure 4:**
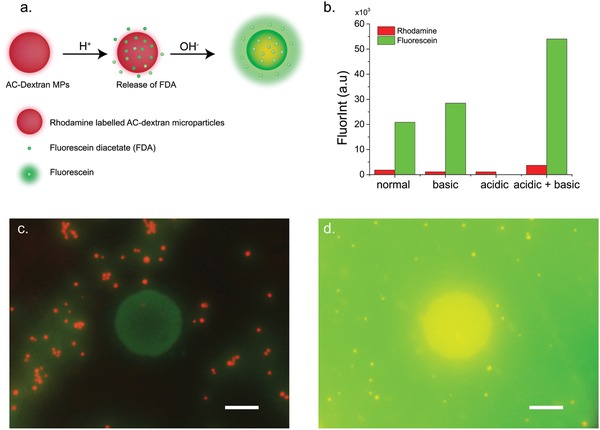
Degradation of acid sensitive microparticles with pH modulator: a) Schematic of dextran‐based acid‐sensitive microparticles. The microparticles decompose in acidic conditions and release FDA. Under basic conditions, FDA hydrolyses and is converted to fluorescein, which in turn shows increased fluorescence. b) Histogram showing fluorescent intensity of microparticles (red) and released (green) fluorescein, in four different pH conditions: normal (pH 7.4), basic (pH 8), acidic (pH 6), acidic + basic (first pH 6, then pH 8). The pH changes were induced by the pH modulator using 8 PdNPs contacts. Six of them with size 400 × 400 µm and two of 1 × 1 mm and openings of 100 µm. c,d) Overlay images showing both red fluorescence (red dots) of rhodamine labeled Ac‐Dextran microparticles and green fluorescence intensity (GFP) of fluorescein released from the particles on top of a PdNPs contact at pH 7.4 and at pH 6 and 8, respectively. Scale bar 50 µm.

To degrade the microparticles, acidic conditions were induced by applying *V*_d_ = 1 V, in the presence of 20 µL of acetalated dextran microparticles (Ac‐Dex MPs). After 180 s, the pH of the solution, which was monitored by a pH microelectrode, reached at pH 6. We incubated the solutions for 24 h in sterile dark conditions, and then we induced basic conditions (pH 8) to hydrolyze FDA. Figure 4b shows the extracted fluorescence intensity of green (FDA‐Fluorescein) and red fluorescence (Rhodamine) for the samples that incubated in the four different pH conditions. The green fluorescence intensity of the samples that were exposed to the acidic then basic conditions was the highest, validating both the degradation of the particles in pH 6 and the subsequent FDA hydrolysis. At pH 7.4, green florescence intensity was observed at the surroundings of the microparticles (Figure 4c). At pH 6, green fluorescence is not detected (Figure S7a, Supporting Information). This is expected for two reasons, FDA is not hydrolyzed and fluorescein does not exhibit fluorescence at acidic pH.^36^, ^37^ At pH 8, there is a higher green fluorescence intensity (Figure S7b, Supporting Information), which indicates that the Ac‐Dex MPs release FDA; however, the intensity is half compared to the samples of interest (Figure 4d). The fluorescence intensity in the samples of interest is the highest, it has a homogenous distribution with highest intensity on top of the electrode surface.

We have developed a Pd based modulator capable of both increasing and decreasing pH in buffered conditions. The pH modulator can increase and decrease pH with an electronic input, *V*_d_. The pH modulator is based on an array of Pd contacts modified with PdNPs to increase surface area and contact capacitance. These contacts are connected to a H^+^ reservoir via a polymer proton conducting bridge. The H^+^ reservoir affords a large supply H^+^ for extended pH modulation. As a proof‐of‐concept, we stimulated the degradation of acid sensitive microparticles, by reducing the solution pH. The microparticles released FDA, a pH sensitive fluorescent dye, and by inducing basic conditions, the fluorescence of the dye was increased. The platform can repetitively control the pH in buffer conditions and can be used for a variety of applications including delivery of cargo to cells^38^ and control of those biochemical reactions in which pH plays an important role.

## Experimental Section

*Device Fabrication*: Micrometer size pH modulator was fabricated using photolithography. Glass slides were sonicated for 20 min in 80% v/v acetone and 20% v/v iso‐propanol (IPA), and dried with N_2_. S1813 (Dow chemicals) photoresist was deposited on top of the glass substrates, following standard protocols (spin‐coated at 3000 rpm, baked 1 min. at 110 °C), to create the Pd patterns. Chromium (Cr) 5 nm and Pd 100 nm were evaporated using an e‐beam evaporator, and a lift‐off process (sonication in 80% v/v Acetone and 20% v/v IPA for 5 min) defined the metal contacts and interconnects. An additional S1813 process defined the area of the Pd contacts for PdNPs deposition, while the metal interconnects were insulated. After PdNP deposition, the photoresist was striped with acetone and the samples were activated with oxygen plasma 100 RF for 2 min.

A blend of 8 wt% polyvinyl alcohol (PVA) with 2 wt% polystyrene sulfonic acid (4:1 weight ratio) was thoroughly mixed (PVA:PSS solution) and sonicated for 45 min. The PVA:PSS solution was filtered with a filter porous size of 0.8 µm and was spin‐coated on top of the samples at 3000 rpm for 30 s and baked in 120 °C for 45 min, yielding a film thickness of 800 nm. A positive photoresist Dow SPR220‐4.5 was spin‐coated following protocols of the manufacturer and patterned to define a pattern for a polyethyleneimine (PEI) membrane which was spin‐coated at 1000 rpm, with a ramp up of 200 rpm s^−1^, for 30 s and then baked at 70 °C for 20 min. The PEI membrane was coated with an additional layer of S1813 (same process) to cover the area on top of the contacts. This layer was patterned with exposure to UV light for 12 s at a power of 8 mW cm^−2^ and developed with MF26A developer for 90 s. The samples were etched with oxygen plasma (O2 15 sccm, Power 200 W for 11 min). A developed monolayer of 3‐glycidoxypropyltrimethoxysilane (GOPS) on top of the glass was formed by chemical vapor deposition under vacuum in 90 °C for 2 h. On top of this was spin‐coated the final insulating layer of SU8‐2005 at 3000 rpm for 30 s.

*Pd Nanoparticles Deposition*: 10 wt% PdNO_3_, purchased from sigma, was diluted with di‐water to give a 1 wt% PdNO_3_ solution. PdNPs were electrochemically deposited onto the Pd contacts using a DC voltage of *V* = −0.6 V with a varied deposition time between 0.1 and 10 s. This resulted in a darkening of the contacts where the NPs were successfully deposited.

*Ag/AgCl Nanoparticles Deposition*: Ag was electrodeposited on top of the Pd contact in the reservoir, by using a solution containing 50 × 10^−3^ m of AgNO_3_ and 0.2 m sulfuric acid in di‐water, by using a constant current of −1 mA for 160 s. Ag/AgCl electrode was used as a RE and Pt wire was used as a counter electrode (CE). Then CV was used to deposit chloride on top of the Ag, by using a solution containing 0.5 m NaCl and 0.2 m HCl in di‐water. Five cycles were carried from −0.45 to 0.9 V with a scan rate from 0.1 V s^−1^ (data not shown).

*PVA:PSS Bridge*: The bridge was created by mixing PVA 10 wt% in di‐water with PSS 30 wt% that resulted in a mass ratio of 4:1 with final PVA concentration 8 wt% and PSS 2 wt%. This 10 wt% PVA (M_w_ ≈ 89000–98000 99% Sigma‐Aldrich) powder was dissolved in di‐water while heating in a microwave oven for a total of 30 s, during which, at 5 s intervals the microwave was paused, and the solution was thoroughly vortexed until clear solution was obtained. Upon continuous mixing, a 30 wt% solution of Na^+^PSS^−^ solution (Sigma Aldrich) was slowly added into the PVA, afterward, the blend solution was sonicated for 45 min. Prior to the deposition the viscous solution was filtered with a commercial polyester (PET) filter of 0.8 µm porous size.

*PEI:APTES Membrane*: To reduce unwanted H^+^ diffusion, a 300 nm thick membrane consisting of PEI cross‐linked with 3‐aminopropyltrimethoxysilane (APTES) at a weight ratio of (1:1) with a final concentration of 3% weight ratio in di‐water was patterned. In physiological and acidic pH, the positively charged amine groups of PEI and APTES repelled positively charged H^+^, acting as a barrier to undesirable H^+^ diffusion. Cross‐linked APTES groups stabilized the film. The positively charged PEI membrane solution was created by mixing polyethyleneimine (PEI: M_w_ ≈ 800 Sigma‐Aldrich) with (APTES at a weight ratio of 1:1 with the final concentration of 3 wt%. This mixture was spin‐coated onto devices at 1000 rpm, with a ramp of 200 rpm s^−1^, for 30 s and then baked at 60 °C for 20 min.

*Characterization*: Device characterization was done utilizing both an Autolab potentiostat and national instruments (NI) PXI with a digital multimeter (DMM) and a source measurement unit (SMU). A custom labview program was controlling the NI system. Cyclic voltammetry (CV) and frequency response analysis (FRA) were performed with the autolab potentiostat.

*Fluorescence Measurements*: All the fluorescence intensity measurements were performed by a Keyence BZX microscope and analyzed with an ImageJ software.

*Synthesis of pH Sensitive Ac‐Dex Microparticles (MPs)*: pH‐sensitive Ac‐Dex was synthesized by following and modifying a previously described method.^27^

*pH‐Triggered MPs Deformation*: 1 mg mL^−1^ Ac‐Dex MPs were dissolved in HEPES 10 × 10^−3^ m buffer (pH = 7.3). The particles were sonicated for 45 min. For each measurement 20 µL of solution was drop casted on top of the device. A negative *V* = −1 V versus Ag/AgCl pellet was applied for 2 min, during which, the pH increased from the initial pH = 7.3 to pH = 8. The pH of the solution was monitored by a micro pH electrode (Fisher Scientific).

## Conflict of Interest

The authors declare no conflict of interest.

## Supporting information

Supplementary

## References

[1] H. Tang , F. Yan , P. Lin , J. Xu , H. L. W. Chan , Adv. Funct. Mater. 2011, 21, 2264.

[2] X. Strakosas , M. Huerta , M. J. Donahue , A. Hama , A. M. Pappa , M. Ferro , M. Ramuz , J. Rivnay , R. M. Owens , J. Appl. Polym. Sci. 2017, 134.

[3] D. Khodagholy , V. F. Curto , K. J. Fraser , M. Gurfinkel , R. Byrne , D. Diamond , G. G. Malliaras , F. Benito‐Lopez , R. M. Owens , J. Mater. Chem. 2012, 22, 4440.

[4] L. Kergoat , B. Piro , D. T. Simon , M. C. Pham , V. Noel , M. Berggren , Adv. Mater. 2014, 26, 5658. 24924118 10.1002/adma.201401608

[5] D. Khodagholy , T. Doublet , P. Quilichini , M. Gurfinkel , P. Leleux , A. Ghestem , E. Ismailova , T. Herve , S. Sanaur , C. Bernard , G. G. Malliaras , Nat. Commun. 2013, 4, 1575. 23481383 10.1038/ncomms2573 PMC3615373

[6] D. Khodagholy , J. N. Gelinas , T. Thesen , W. Doyle , O. Devinsky , G. G. Malliaras , G. Buzsaki , Nat. Neurosci. 2015, 18, 310. 25531570 10.1038/nn.3905 PMC4308485

[7] L. H. Jimison , S. A. Tria , D. Khodagholy , M. Gurfinkel , E. Lanzarini , A. Hama , G. G. Malliaras , R. M. Owens , Adv. Mater. 2012, 24, 5919. 22949380 10.1002/adma.201202612

[8] M. Ramuz , A. Hama , M. Huerta , J. Rivnay , P. Leleux , R. M. Owens , Adv. Mater. 2014, 26, 7083. 25179835 10.1002/adma.201401706 PMC4489338

[9] S. F. Cogan , Annu. Rev. Biomed. Eng. 2008, 10, 275. 18429704 10.1146/annurev.bioeng.10.061807.160518

[10] J. Delbeke , Biocybern. Biomed. Eng. 2011, 31, 81.

[11] R. S. Fisher , Ann. Neurol. 2012, 71, 157. 22367987 10.1002/ana.22621 PMC3296971

[12] A. Williamson , J. Rivnay , L. Kergoat , A. Jonsson , S. Inal , I. Uguz , M. Ferro , A. Ivanov , T. A. Sjostrom , D. T. Simon , M. Berggren , G. G. Malliaras , C. Bernard , Adv. Mater. 2015, 27, 3138. 25866154 10.1002/adma.201500482

[13] J. Isaksson , P. Kjall , D. Nilsson , N. D. Robinson , M. Berggren , A. Richter‐Dahlfors , Nat. Mater. 2007, 6, 673. 17643105 10.1038/nmat1963

[14] D. T. Simon , S. Kurup , K. C. Larsson , R. Hori , K. Tybrandt , M. Goiny , E. W. Jager , M. Berggren , B. Canlon , A. Richter‐Dahlfors , Nat. Mater. 2009, 8, 742. 19578335 10.1038/nmat2494

[15] A. Jonsson , S. Inal , I. Uguz , A. Williamson , L. Kergoat , J. Rivnay , D. Khodagholy , M. Berggren , C. Bernard , G. G. Malliaras , D. T. Simon , Proc. Natl. Acad. Sci. U. S. A. 2016, 113, E6903. 27506784 10.1073/pnas.1604231113 PMC5003234

[16] T. E. DeCoursey , J. Physiol. 2008, 586, 5305. 18801839 10.1113/jphysiol.2008.161703 PMC2655391

[17] J. A. Wemmie , R. J. Taugher , C. J. Kreple , Nat. Rev. Neurosci. 2013, 14, 461. 23783197 10.1038/nrn3529 PMC4307015

[18] V. S. Stoll , J. S. Blanchard , Methods Enzymol. 1990, 182, 24. 2314240 10.1016/0076-6879(90)82006-n

[19] P. D. Boyer , B. Chance , L. Ernster , P. Mitchell , E. Racker , E. C. Slater , Annu. Rev. Biochem. 1977, 46, 955. 18361775 10.1146/annurev.bi.46.070177.004515

[20] M. Capasso , T. E. DeCoursey , M. J. S. Dyer , Trends Cell Biol. 2011, 21, 20. 20961760 10.1016/j.tcb.2010.09.006 PMC3014425

[21] R. A. Duquette , S. Wray , Pflügers Arch. ‐ Eur. J. Physiol. 2001, 442, 459. 11484779 10.1007/s004240100562

[22] M. A. Bumke , D. Neri , G. Elia , Proteomics 2003, 3, 675. 12748947 10.1002/pmic.200300395

[23] Y. Kato , S. Ozawa , C. Miyamoto , Y. Maehata , A. Suzuki , T. Maeda , Y. Baba , Cancer Cell Int. 2013, 13, 89. 24004445 10.1186/1475-2867-13-89 PMC3849184

[24] M. Chesler , Physiol. Rev. 2003, 83, 1183. 14506304 10.1152/physrev.00010.2003

[25] A. E. Ziemann , M. K. Schnizler , G. W. Albert , M. A. Severson , M. A. Howard 3rd , M. J. Welsh , J. A. Wemmie , Nat. Neurosci. 2008, 11, 816. 18536711 10.1038/nn.2132 PMC2553357

[26] S. Joshi‐Barr , C. D. Lux , E. Mahmoud , A. Almutairi , Antioxid. Redox Signaling 2014, 21, 730. 10.1089/ars.2013.5754 PMC4098119 24328819

[27] A. Almutairi , S. J. Guillaudeu , M. Y. Berezin , S. Achilefu , J. M. J. Frechet , J. Am. Chem. Soc. 2008, 130, 444. 18088125 10.1021/ja078147e

[28] J. Isaksson , D. Nilsson , P. Kjall , N. D. Robinson , A. Richter‐Dahlfors , M. Berggren , Org. Electron. 2008, 9, 303. 10.1038/nmat1963 17643105

[29] Y. Deng , T. Miyake , S. Keene , E. E. Josberger , M. Rolandi , Sci. Rep. 2016, 6, 24080. 27052724 10.1038/srep24080 PMC4823714

[30] T. Miyake , E. E. Josberger , S. Keene , Y. Deng , M. Rolandi , APL Mater. 2015, 3, 014906.

[31] K. Tybrandt , K. C. Larsson , S. Kurup , D. T. Simon , P. Kjäll , J. Isaksson , M. Sandberg , E. W. H. Jager , A. Richter‐Dahlfors , M. Berggren , Adv. Mater. 2009, 21, 4442.

[32] E. E. Josberger , Y. Deng , W. Sun , R. Kautz , M. Rolandi , Adv. Mater. 2014, 26, 4986. 24789251 10.1002/adma.201400320

[33] T. Imokawa , K. J. Williams , G. Denuault , Anal. Chem. 2006, 78, 265. 16383336 10.1021/ac051328j

[34] J. Horkans , J. Electroanal. Chem. Interfacial Electrochem. 1986, 209, 371.

[35] M. Grden , M. Lukaszewski , G. Jerkiewicz , A. Czerwinski , Electrochim. Acta 2008, 53, 7583.

[36] T. J. Battin , Sci. Total Environ. 1997, 198, 51.

[37] M. J. Doughty , S. Glavin , Ophthalmic Physiol. Opt. 2009, 29, 573. 19686307 10.1111/j.1475-1313.2009.00683.x

[38] Z. Hemmatian , E. jalilian , S. Lee , X. Strakosas , A. Khademhosseini , A. Almutairi , S. Shin , M. Rolandi , ACS Appl. Mater. Interfaces 2018, 10, 21782. 29905062 10.1021/acsami.8b02724

