# Supplementary material for: A Bioelectronic Platform Modulates pH in Biologically Relevant Conditions
Source: Adv Sci (Weinh). 2019 Jan 28;6(7):1800935. doi: 10.1002/advs.201800935 (PMC6446605; doi:10.1002/advs.201800935)
Supplement: Supplementary file 1 — Supplementary [file ADVS-6-1800935-s001.pdf]

Copyright WILEY-VCH Verlag GmbH & Co. KGaA, 69469 Weinheim, Germany, 2018.

Supporting Information

for *Adv. Sci.,* DOI: 10.1002/advs.201800935

A Bioelectronic Platform Modulates pH in Biologically Relevant Conditions

*Xenofon Strakosas, John Selberg, Xiaolin Zhang, Noah Christie, Peng-Hao Hsu, Adah Almutairi, and Marco Rolandi**

Supplementary information

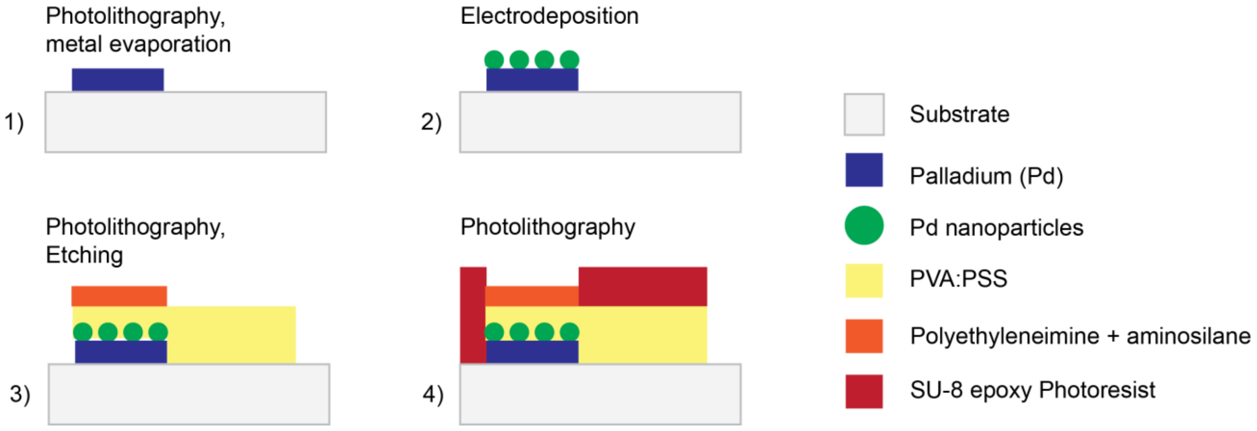

Figure S1: fabrication process of pH modulator. Step 1) photolithography and Palladium thin film evaporation. Step 2: photolithography for defining the Pd electrode and electrodeposition of Pd to create Pd nanoparticles. Step 3: photolithography to define the PVA:PSS bridge and the PEI:APTES selective membrane with subsequent O2 etching. Step 4: photolithography of SU8 photoresist to seal the interconnects and define the contact with the electrolyte.

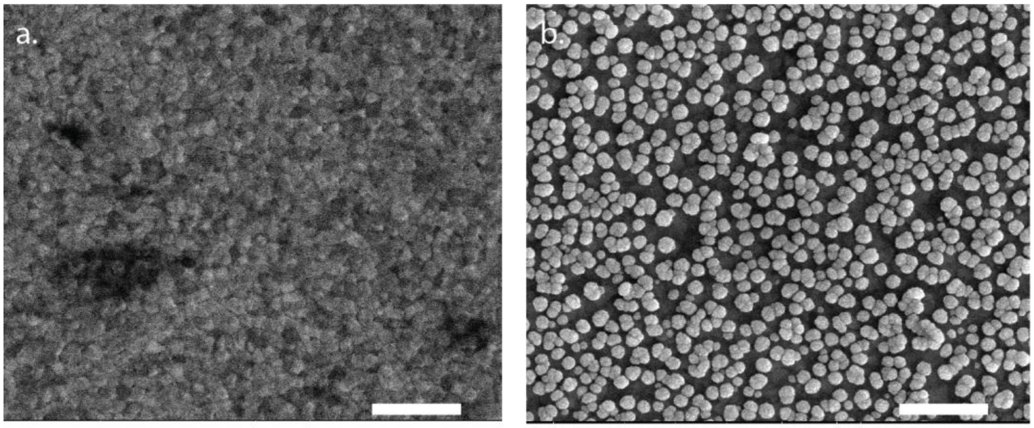

Figure S2: SEM images between a. planar Pd and b. PdNPs modified contact. scale bar 500 nm

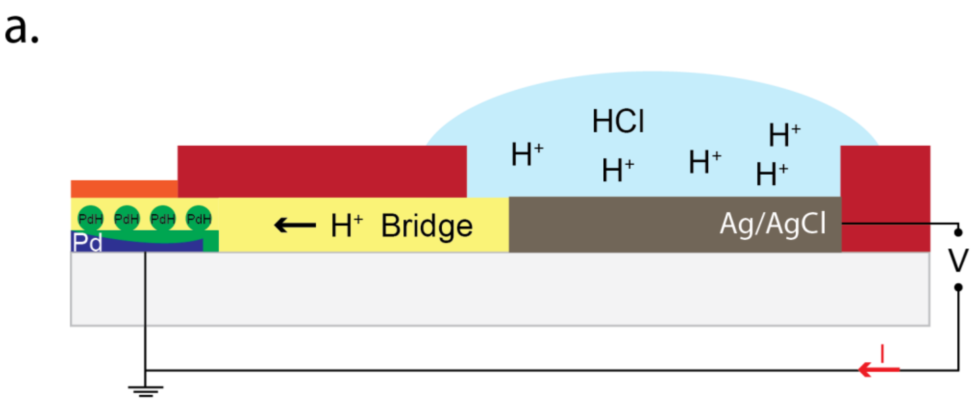

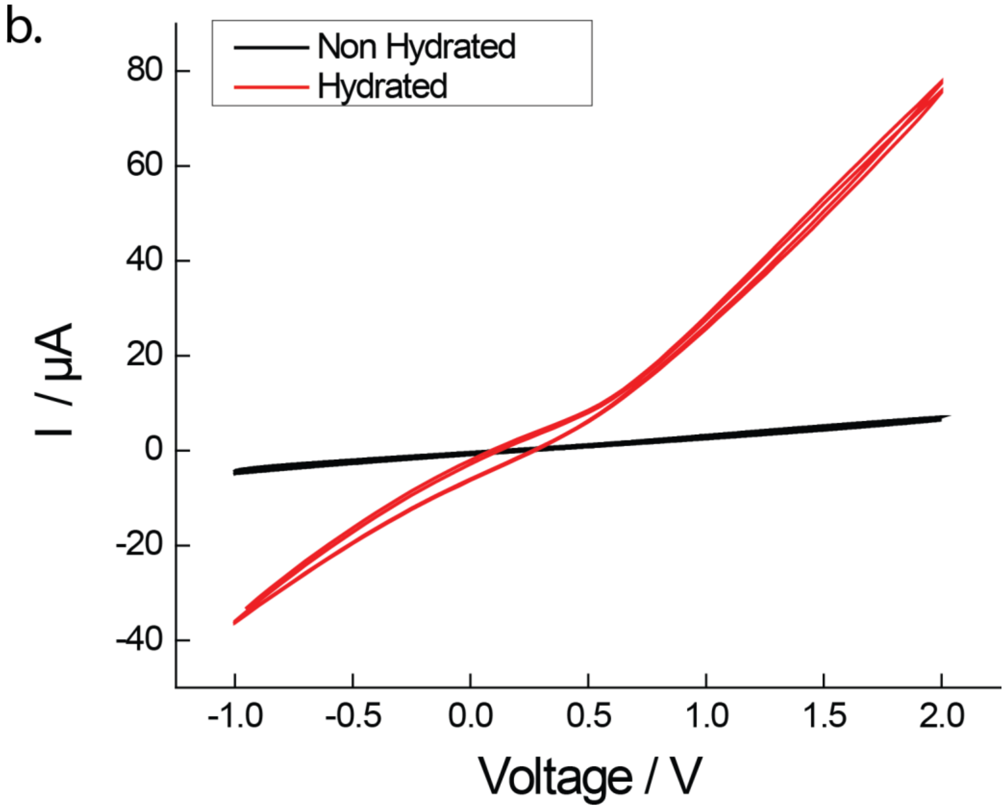

Figure S3: : improved performance after hydration a) Schematic showing the applied voltage (V) between PdNPs electrode and external AgCl electrode b) Cyclic voltammetry for Vt before (black) and after the device was hydrated in di-water for 24 hours.

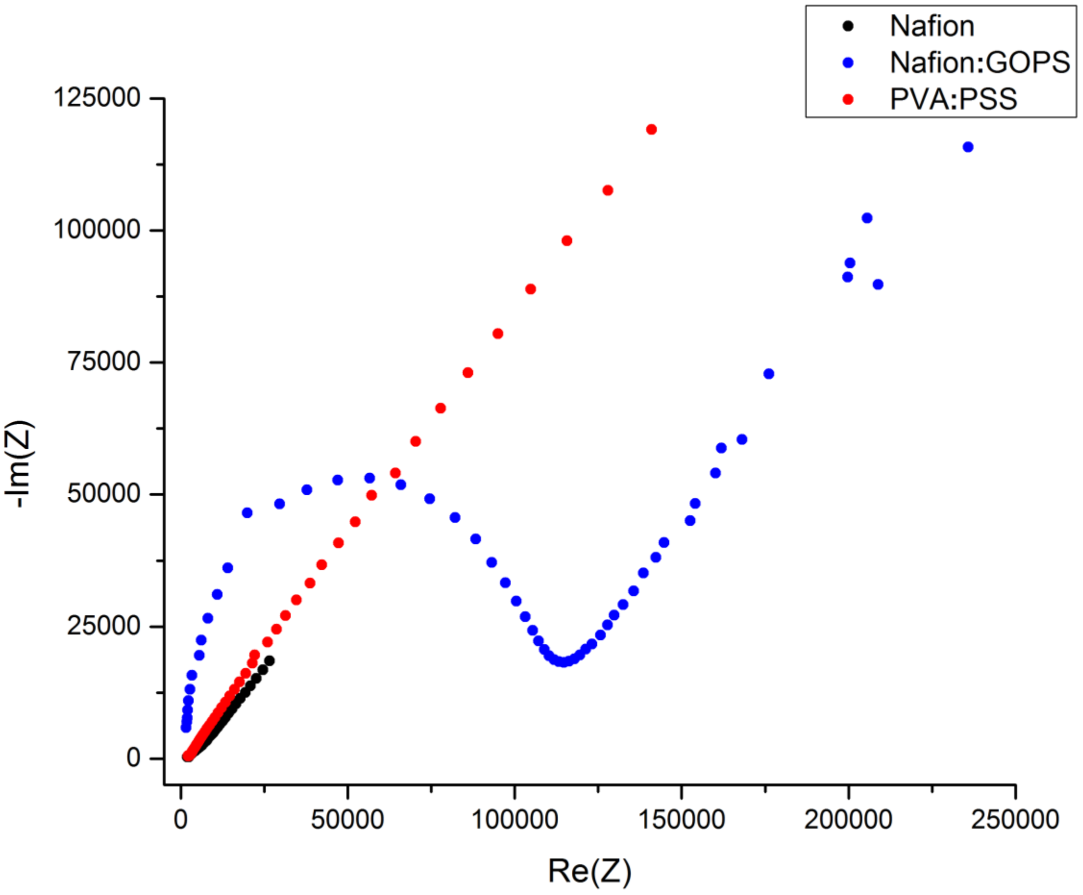

Figure S4: : Nyquist plots of Nafion 117 (black), PVA:PSS (red), and Nafion:GOPS (blue) thin films. The films were patterned on top of the Au metal electrodes with dimensions (width 2cm and length 50 um)

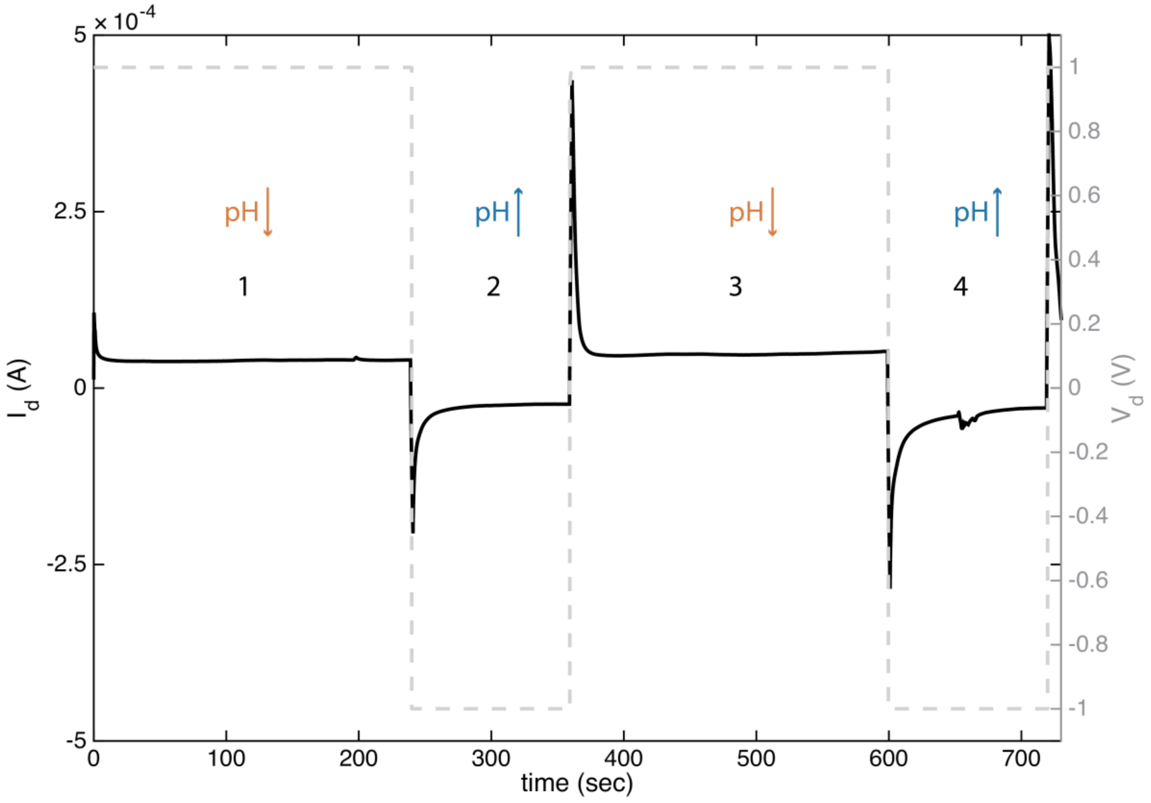

Figure S5: pH modulator creating initially acidic pH. When PdNPs have not absorbed H^+ into their structure (pure Pd), the current is mainly resistive with a small initial capacitance (curve 1). The initial capacitance can be attributed to the OH^- adsorption onto the PdNPs surface. After loading the PdNPs with H^+ (curve 2), the current has a higher initial capacitance (curve 3) due to the combination of H^+ release from PdH_x and subsequent OH^- adsorption.

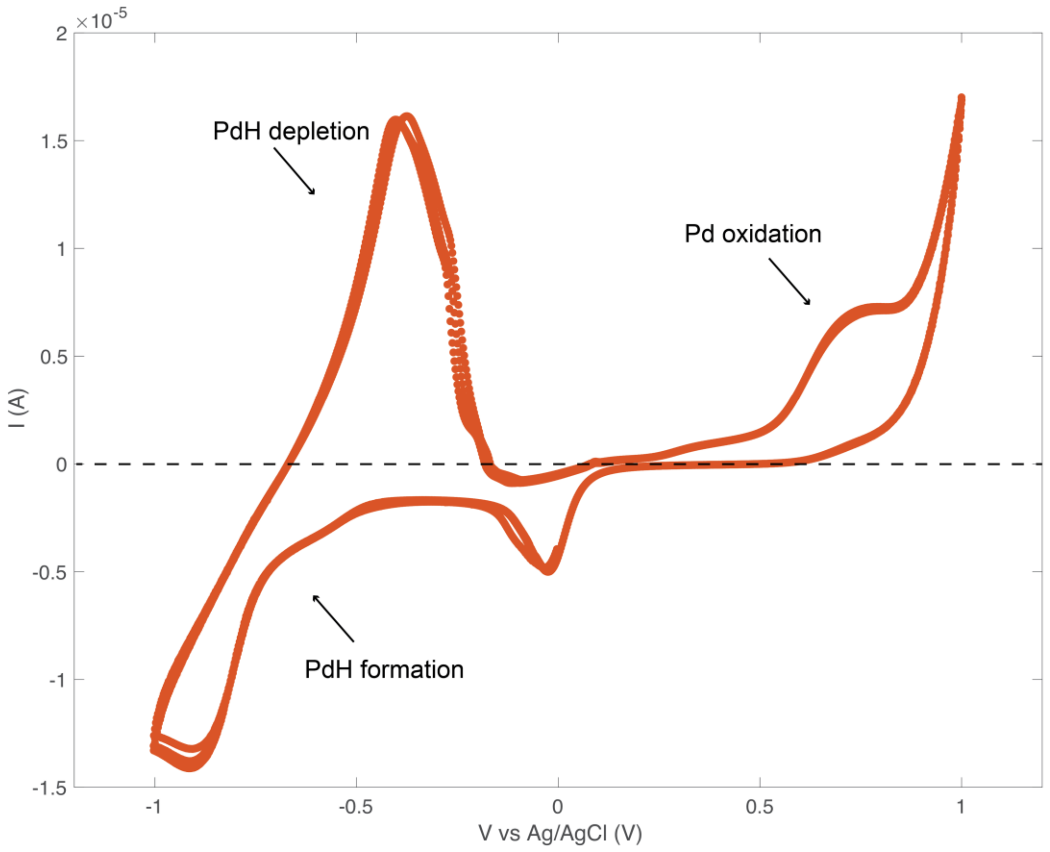

Figure S6: Behavior of PdNPs contact in positive potential. CV shows an oxidative current from starting from 0.7 V until 1V(orange). This current is attributed to the OH^- adsorbing on the surface of the Pd.

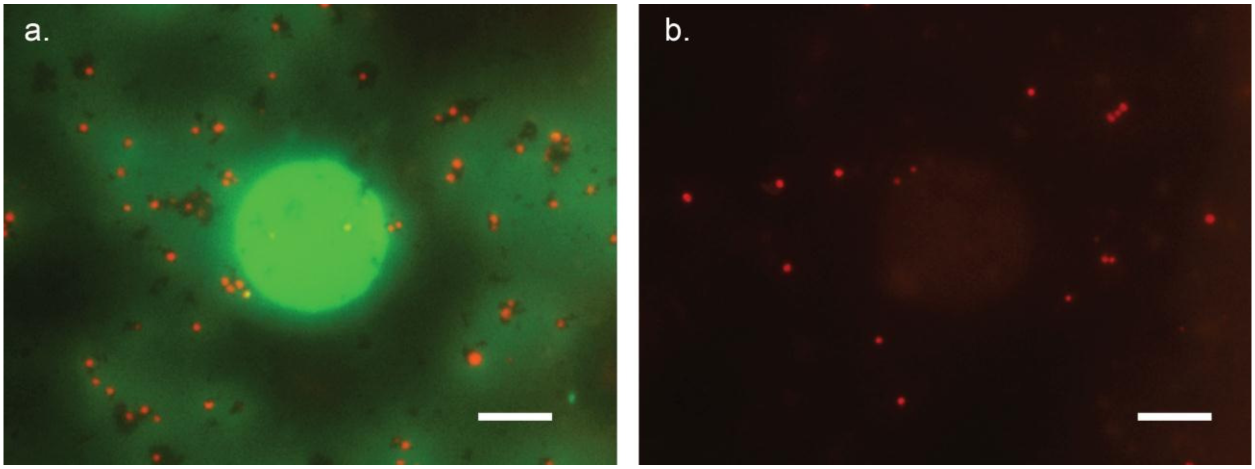

Figure S7: Acid sensitive microparticles degradation. a) Overlay red fluorescence (red dots) of rhodamine labelled Ac-Dextran micro-particles and green fluorescence (GFP) of fluorescein released from the particles on top of a pore of a 100 um PdNPs contact in pH 8. pH was increased by the PdNPs contact upon a V_d = -1 V vs an Ag/AgCl pellet. b) Overlay red fluorescence (red dots) of rhodamine labelled Ac-Dextran micro-particles and green fluorescence intensity (GFP) of fluorescein released from the particles in pH 6 on top of the opening of a 100 um PdNPs contact. pH was decreased by upon a V = 1 V vs an Ag/AgCl pellet. Scale bars 50 um.
